# Supplementary material for: A DERIVED RELAXATION CONTRAST FROM SYNTHETIC MRI FOR DETECTING NETWORK MICROSTRUCTURAL VULNERABILITY
Source: bioRxiv. 2026 Apr 12:2026.04.08.717271. Preprint. [Version 1] doi: 10.64898/2026.04.08.717271 (PMC13082010; doi:10.64898/2026.04.08.717271)
Supplement: Supplement 1 [file media-1.pdf]

SEMI-QUANTITATIVE CONTRAST FOR MYELIN/LIPID QUANTIFICATION USING SYNTHETIC FLAIR AND DIR IN MCI

Anupa Ekanayake[1], Scott Hwang[1], Senal Peiris[1], Rommy Elyan[1], Mustafa Ghulam[1], Mark Tulchinsky[1], Jianli Wang[1], Paul Eslinger[1, 2], Qing Yang[1, 3], and Prasanna Karunanayaka[1]

SUPPLEMENTARY MATERIAL

Running title: Myelin changes in MCI

Author affiliations:

[1]Department of Radiology, Penn State University College of Medicine, Hershey, PA, USA

[2]Department of Neurology, Penn State University College of Medicine, Hershey, PA, USA

[3]Department of Neurosurgery, Penn State University College of Medicine, Hershey, PA, USA

FLAIR signal model

Using standard approximation:

$$S_{\text{FLAIR}} \approx M_0\left(1 - 2e^{-TI_F/T1} + e^{-TR/T1}\right)e^{-TE_F/T2}$$

let

$$A_F(T1) = \left(1 - 2e^{-TI_F/T1} + e^{-TR/T2}\right)$$

then

$$S_{\text{FLAIR}} \approx M_0\, A_F(T1)\, e^{-TE_F/T2}.$$

DIR signal model (double inversion)

A common simplified DIR approximation is:

$$S_{\text{DIR}} \approx M_0\left(1 - 2e^{-TI_1/T1} + 2e^{-TI_2/T1} - e^{-TR/T1}\right)e^{-TE_D/T2}$$

Define:

$$A_D(T_1) = \left(1 - 2e^{-TI_1/T1} + 2e^{-TI_2/T1} - e^{-TR/T1}\right)$$

then

$$S_{\text{DIR}} \approx M_0\, A_D(T1)\, e^{-TE_D/T2}.$$

Similarly, DF definition:

$$FD = \frac{\text{FLAIR} - \text{DIR}}{\text{FLAIR}} = 1 - \frac{\text{DIR}}{\text{FLAIR}} = 1 - \frac{S_{\text{DIR}}}{S_{\text{FLAIR}}}.$$

Substitute respective signal models:

$$FD = 1 - \frac{M_0\, A_D(T1)\, e^{-TE_D/T2}}{M_0\, A_F(T1)\, e^{-TE_F/T2}}$$

$$FD = 1 - \frac{A_D(T1)}{A_F(T1)}\ e^{-(TE_D - TE_F)/T2}.$$

The closed-form DF expression is:

$$FD = 1 - \frac{\left(1 - 2e^{-TI_1/T1} + 2e^{-TI_2/T1} - e^{-TR/T1}\right)}{\left(1 - 2e^{-TI_F/T1} + e^{-TR/T1}\right)}\ e^{-(TE_D - TE_F)/T2}$$

Conceptual interpretation of FD

The FD metric is essentially measuring differences in inversion-recovery contrast between FLAIR and DIR which are governed by:

- tissue T1 recovery
- T2 decay
- myelin / lipid microstructure

FD therefore behaves like a derived relaxation contrast reflecting network microstructural integrity

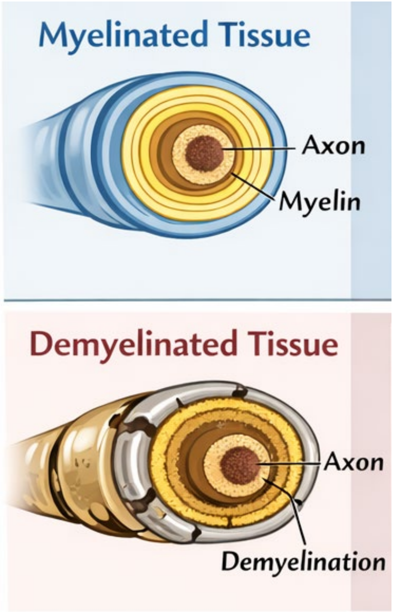

Figure S1. Conceptual illustration of low and high FD values in MCI. Variations in tissue microstructure may differentially affect FLAIR and DIR signals, leading to either reduced or increased FD values depending on the nature and stage of underlying microstructural alterations.

.

Mathematical Meaning T1/T2 ratio

T1= longitudinal (spin-lattice) relaxation time

T2= transverse (spin-spin) relaxation time

Both are measured in milliseconds, so the units cancel, making the ratio dimensionless.

Tissue	T1 (ms)	T2 (ms)	T1/T2
White matter	~800	~80	~10
Gray matter	~1300	~100	~13
CSF	~4000	~2000	~2

Different tissues have different T1/T2 ratios, which contributes to MRI contrast. It is sensitive to:

- Myelin disruption
- lipid changes
- microstructural alterations

The relationship between FD and T1/T2 Ratio

Tissue Property	Effect
Myelin	Short T1, short T2
Lipid content	Alters both
Water content	Long T1, long T2

Microstructural disruption changes both relaxation times

Therefore:

$$DF = f(T1, T2)$$

which indirectly relates to the T1/T2 ratio, a known myelin-sensitive contrast. Therefore, FD can be considered sensitive to tissue properties that modulate effective $T1/T2$—including myelin- and lipid-related microstructural alterations—while remaining scalable because it is computed from routinely generated synthetic contrasts.
